# Role of airway smooth muscle cell phenotypes in airway tone and obstruction in guinea pig asthma model

**DOI:** 10.1186/s13223-022-00645-7

**Published:** 2022-01-11

**Authors:** Mayra D. Álvarez-Santos, Marisol Álvarez-González, Elizabeth Eslava-De-Jesus, Angel González-López, Ivonne Pacheco-Alba, Yazmín Pérez-Del-Valle, Rodrigo Rojas-Madrid, Blanca Bazán-Perkins

**Affiliations:** 1 Biology Area, Facultad de Ciencias, Universidad Nacional Autónoma de México, 04510 Mexico City, Mexico; 2 Laboratorio de Inmunofarmacología, Instituto Nacional de Enfermedades Respiratorias Ismael Cosío Villegas, 14080 Mexico City, Mexico; 3 Tecnologico de Monterrey, Escuela de Medicina y Ciencias de la Salud, 14380 Mexico City, Mexico

**Keywords:** Airway smooth muscle, Airway obstruction, TGF-β1, GSH, SERCA, Airway responsiveness, Airway tone

## Abstract

**Background:**

Airway obstruction (AO) in asthma is driven by airway smooth muscle (ASM) contraction. AO can be induced extrinsically by direct stimulation of ASM with contractile agonists as histamine, or by indirect provocation with antigens as ovalbumin, while the airway tone is dependent on intrinsic mechanisms. The association of the ASM phenotypes involved in different types of AO and airway tone in guinea pigs was evaluated.

**Methods:**

Guinea pigs were sensitized to ovalbumin and challenged with antigen. In each challenge, the maximum OA response to ovalbumin was determined, and before the challenges, the tone of the airways. At third challenge, airway responsiveness (AR) to histamine was evaluated and ASM cells from trachea were disaggregated to determinate: (a) by flow cytometry, the percentage of cells that express transforming growth factor-β1 (TGF-β1), interleukin-13 (IL-13) and sarco-endoplasmic Ca^2+^ ATPase-2b (SERCA2b), (b) by RT-PCR, the SERCA2B gene expression, (c) by ELISA, reduced glutathione (GSH) and, (d) Ca^2+^ sarcoplasmic reticulum refilling rate by microfluorometry. Control guinea pig group received saline instead ovalbumin.

**Results:**

Antigenic challenges in sensitized guinea pigs induced indirect AO, AR to histamine and increment in airway tone at third challenge. No relationship was observed between AO induced by antigen and AR to histamine with changes in airway tone. The extent of antigen-induced AO was associated with both, TGF-β1 expression in ASM and AR degree. The magnitude of AR and antigen-induced AO showed an inverse correlation with GSH levels in ASM. The airway tone showed an inverse association with SERCA2b expression.

**Conclusions:**

Our data suggest that each type of AO and airway tone depends on different ASM phenotypes: direct and indirect AO seems to be sensitive to the level of oxidative stress; indirect obstruction induced by antigen appears to be influenced by the expression of TGF-β1 and the SERCA2b expression level plays a role in the airway tone.

## Introduction

Airway smooth muscle (ASM) is a central structure involved in the development of asthma physiopathology since it contributes to the development of airway hyperresponsiveness (AHR) and variable airflow obstruction. In addition, ASMs have a persistent contractile force capable of maintaining a high airway intrinsic baseline tone, which is increased in asthma patients [1, 2]. Notably, it has been suggested that the airway basal tone is an underlying contributor to the airway contractile capacity and AHR through tonic activation of ASM [3–5].

It has been recognized that ASM is not only a primary contributor to the development of physiological alterations in asthma but also induces important airway environmental changes [6]. For example, sensitization of ASM with IgE upregulated the expression of interleukin-13 (IL-13) in isolated rabbit trachea via a process that enhances the contractility of ASM due to autocrine effects [7]. In small isolated airways from humans, IL-13 can induce AHR [8], and IL-13 can increase both the baseline airway tone and the airway responsiveness in murine lung slices [3]. Another example is transforming growth factor β1 (TGF-β1), a cytokine that is produced at high levels by ASM cells in asthma. TGF-β1 is a potent profibrogenic factor involved in multiple cellular responses, such as differentiation, proliferation and survival [9]. Although the effects of TGF-β1 on airway contractility are controversial, it is recognized that this cytokine can be involved in airway remodelling by inducing ASM proliferation and fibrosis and increasing airway wall mass [9, 10]. It has been revealed that changes in airway basal tone can also be attributed to airway wall enlargement induced by structural changes [11].

Another factor that can induce changes in airway physiology is oxidative stress. Reduced glutathione (GSH) is a thiol antioxidant that is highly expressed in the epithelial lining fluid and within airway cells, where it acts as a regulator of the cell cycle and enzyme substrates. When it becomes oxidized, GSH forms glutathione disulphide (GSSG), which can inhibit the antioxidant capabilities of the airways through depletion of the total GSH pool [12]. Such an interaction promotes histone acetylation by increasing histone acetyltransferase activity and inhibiting histone deacetylase activity in airway cells, leading to the enhanced release of proinflammatory cytokines [13]. In addition, GSH relaxes the trachea and suppresses AHR to histamine by activating ASM potassium channels [14]. GSH reduces the airway tone, unlike oxidative molecules, which have been shown to be related to increased airway tone and AHR [15].

ASM contraction occurs through an increase in intracellular calcium (Ca^2+^) levels. The baseline Ca^2+^ levels in ASM are maintained by the constant removal of Ca^2+^ either by pumps present on the plasma membrane or by the sarcoendoplasmic reticulum Ca^2+^ ATPase (SERCA). Of the three isoforms, SERCA2b is found in smooth muscles. Indeed, in ASM cells from asthma patients, AHR has shown to be related to downregulation of the function and expression of SERCA2b [16]. Until now, the role of SERCA2b in airway baseline tone has been unknown.

Guinea pigs have been a valuable tool in the study of human asthma. Direct stimulation of ASM with agonists such as histamine or indirect provocation with antigens in guinea pigs produce robust airway obstruction because guinea pigs have a large ASM mass [17]. In addition to the extrinsic responses induced by direct and indirect provocation, the guinea pig asthma model has shown an increase in intrinsic baseline tone [18].

It has been found that individual smooth muscle cells can develop changes in their phenotype after physiological and pathological stimulation [19]. In canine trachea, two populations of myocyte subsets have been observed that show differences in contractile phenotype protein abundances and proliferative capacities [20].

Our aim was to identify the phenotype of ASM cells that is related to intrinsic and extrinsic airway obstruction and the relationship between the different types of airway obstruction in an asthma model in guinea pigs.

## Materials and methods

### Study design

**Fig. 1:**
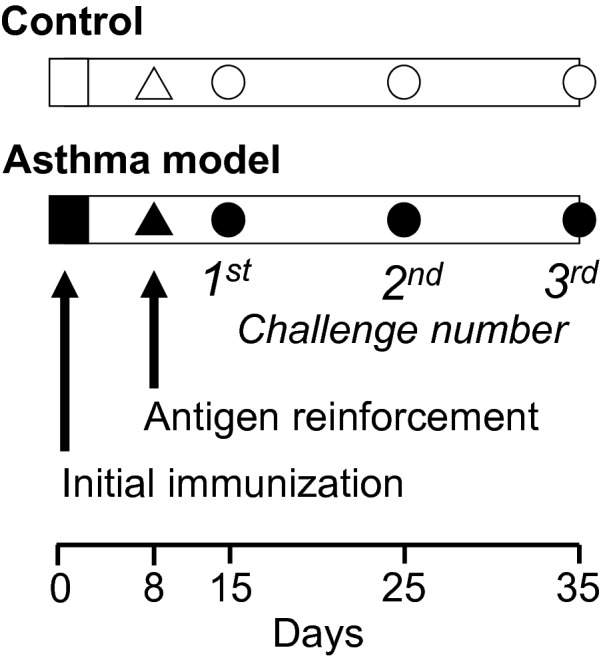
Experimental design. Guinea pigs were sensitized by intraperitoneal (0.5 mg/ml) and subdermal (0.5 mg/ml) injections with a combination of ovalbumin (60 μg/ml) and 1 mg/ml aluminium hydroxide dispersed in physiological saline solution (black squares). Eight days later, the sensitization was reinforced with ovalbumin aerosol administration for 5 min (3 mg/ml; black triangle). From day 15 onward, guinea pigs were challenged for one minute with ovalbumin aerosol every 10 days; 1 mg/ml was used for the first challenge, and 0.5 mg/ml was used for the subsequent challenges (black circles). At the third challenge, dose–response curves in response to histamine were generated, and tissue acquisition was performed. The animals in the control group (open figures) received physiological saline solution instead of ovalbumin

As described in Fig. 1, after the initial sensitization, guinea pigs were intermittently exposed to aerosolized ovalbumin via up to three antigenic challenges. At the last challenge, the development of antigen-induced airway responsiveness was evaluated by generating dose–response curves showing the response to histamine before and after an antigenic challenge. Afterward, the animals were sacrificed to obtain lung and trachea samples. In the lung samples, areas in the lamina propria and the ASM layer were analysed through light microscopy. ASM cells were disaggregated from the trachea, and flow cytometry was used to determine the percentages of cells that expressed TGF-β1, IL-13 and SERCA2b. SERCA2B gene expression was determined in isolated ASM cells by real-time polymerase chain reaction (RT-PCR). The level of GSH in ASM cells was measured by enzyme-linked immunosorbent assay (ELISA). Statistical analysis of the association of each parameter in ASM cells with values related to extrinsic and intrinsic airway obstruction was performed using Spearman correlation coefficient analysis. Some ASM cells were loaded with Fura-2 acetoxymethyl ester (Fura-2/AM), and the sarcoplasmic reticulum Ca^2+^ refilling rate after caffeine withdrawal was evaluated by microfluorometry. Control animals received sham treatment with saline solution.

### Asthma model

Guinea pigs were sensitized and challenged with ovalbumin (asthma model) or saline (control) solutions according to previously described methods [17] (Fig. 1). Antigen or saline nebulization was performed in acrylic chambers, and pulmonary function was recorded by using a whole-body single-chamber plethysmograph for freely moving animals (Buxco Electronics Inc., Troy, NY, USA). The signal from the chamber was processed with the included software (Buxco Bio System XA v1.1) to calculate several respiratory parameters, including the bronchoobstructive index (Bi). This index was determined using the following equation:

$${\text{Bi}} = \left( {\left( {{\text{Te}} - {\text{Rt}}} \right)/{\text{Rt}}} \right) \, \left( {{\text{PEP}}/{\text{PIP}}} \right),$$

where Te = expiratory time (s), Rt = relaxation time (s), PEP = peak expiratory pressure (cmH_2_O), and PIP = peak inspiratory pressure (cmH_2_O).

The software was adjusted to include only breaths with a tidal volume of 1 ml or more in the analysis, with a minimal inspiratory time of 0.15 s, a maximal inspiratory time of 3 s, and a maximal difference between the inspiratory and expiratory volumes of 10%. After the guinea pig was placed inside the plethysmographic chamber, a 5 min baseline Bi recording was initiated 10 min later. Starting one minute after aerosol administration, the Bi was recorded at 5 and 10 min and then every 15 min thereafter. Because the Bi was calculated for each breath, adjustments were made in the software to determine the average values for all breaths occurring over a 15 s interval and then to aver-age these values during the last 5 min of each period.

Aerosols containing ovalbumin or saline were produced by a US-1 Bennett nebulizer (flow, 2 ml/min; Multistage liquid impinger, Burkard Manufacturing Co., Rickmansworth, Hertfordshire, UK), which released a mixture of particles with sizes of < 4 µm (44%), 4–10 µm (38%), and > 10 µm (18%).

Airway responsiveness was evaluated at the third antigenic challenge by the administration of non-cumulative doses of histamine (0.001 to 0.1 mg/ml) aerosol before and after antigen or saline exposure. The histamine doses were delivered over 1 min, and the Bi over the following 10 min was determined. The interval between histamine doses was 10 min. The dose–response curve was complete when the Bi reached three times its baseline level. The second histamine curve was determined three hours after the antigen or saline challenge.

### Isolation of tracheal smooth muscle cells

One hour after completing the second histamine curve, guinea pigs were subjected to an overdose of an intraperitoneal injection of pentobarbital sodium (65 mg/kg), and the trachea and left lung lobe were obtained. Airway smooth muscle that was free of epithelium and connective tissue was dissected and incubated for 10 min at 37 °C in 5 ml of Hanks’ solution (Gibco, Grand Island, NY, USA) containing 2 mg of cysteine and 0.05 U/ml papain. The tissue was washed in Leibovitz’s solution and placed in physiological saline solution containing (mM): 25 NaHCO_3_, 118 NaCl, 1.2 KH_2_PO_4_, 4.6 KCl, 1.2 MgSO_4_, and 11 glucose. The smooth muscle was cut into small strips (5 × 0.5 mm) weighing 200 mg total and placed in 2.5 ml physiological saline solution containing collagenase type I (1 mg/ml; Boehringer-Mannheim, Indianapolis, IN, USA) and dispase II (4 mg/ml) for 10 min at 37 °C. This procedure was repeated twice. Leibovitz’s solution was added to stop the enzymatic activity, and the tissue was dispersed mechanically until isolated cells were observed.

### Flow cytometry

Isolated smooth muscle cells were incubated with 10 g/ml brefeldin-A for 4 h at 37 °C to inhibit cytokine release. Then, the cells were fixed with 4% *p*-formaldehyde for 10 min at 4 °C, washed, and permeabilized with 0.1% saponin, 10% BSA and 1% NaN_3_ in PBS. The cells then underwent gentle shaking in the dark for 15 min at room temperature and were labelled with surface marker antibodies (1 µl/1 × 106 cells) against SERCA2b (clone 2A7-A1, mouse monoclonal SERCA2b ATPase, Abcam, Cambridge, MA, USA), IL-13 (human monoclonal, labeled with APC; BD Biosciences, San Diego, CA, USA) and TGF-β1 (clone 9016, labeled with phycoerythrin; R&D Systems, Minneapolis, MN, USA). Another incubation was performed with the secondary antibody conjugated to FITC (BD Biosciences Pharmingen, San Diego, CA, USA) for 30 min. Then, the cells were analysed for marker expression with a FACScan flow cytometer (Becton Dickinson, San Jose, CA, USA) using CellQuest software, and 10,000 events were counted. To analyse the staining, the blasts were first gated according to their physical properties (forward and side scatter). Next, a second gate was drawn based on the fluorescence characteristics of the gated cells. Subsequently, the percentage of myocytes expressing the marker was determined. Control staining was performed using fluorochrome-conjugated isotype-matched antibodies. Background staining contributed to < 1% of the total signal and was subtracted from the experimental values.

### RT quantitative PCR

Total RNA was extracted from tracheal smooth muscle strips obtained from guinea pigs using TRIzol reagent (Life Technologies, Grand Island, NY, USA). The RNA quality was assessed by resolving the fragments on denatured 1% agarose gels and measurement of the absorbance ratios at 260/280 nm. Total RNA (1 mg) was reverse-transcribed using Moloney murine leukaemia virus reverse transcriptase and 2 μg of random primers according to the manufacturer’s protocol (Advantage RT-for-PCR Kit; Clontech, Palo Alto, CA, USA). Quantitative RT-PCR amplification was performed using the iCycler iQ Detection System (Bio-Rad, Hercules, CA, USA). PCR was performed with the cDNA working mixture in a 20 μl reaction volume containing 20 mM Tris–HCl, 2 μl of cDNA, 200 μM dNTP, 2 mM MgCl_2_, 50 mM KCl, 1 μM each of the specific 5′ and 3′ primers, 1.25 U of Taq DNA polymerase (Roche, Branchburg, NJ, USA) SYBR green (1:50,000) and 10 nM fluorescein (Roche, Indianapolis, IN, USA) at pH 8.3. The primer pair used for SERCA2b amplification was designed in Primer BLAST. The sequences were 5′-TTAAAGCAACTGTCTATTTCTGCTG-3′ and 5′-AGTCAGAAAAAGCAAAACAAAATCTA-3′ (Merck KGaA, Darmstadt, Germany). The PCR cycling conditions consisted of 95 °C for 10 min followed by 40 cycles of 95 °C for 15 s and 60 °C for 1 min. The 18S gene was used as an endogenous gene to normalize the RNA expression (Applied Biosystems® Eukaryotic 18S rRNA Endogenous Control (FAM™ MGB Probe, Non-Primer Limited), Thermo Fisher Scientific, USA). After 40 cycles, the delta-Ct method was used to compare the levels of the transcripts. The results were calculated as 2 elevated to the negative power of the difference between the CT value of each gene minus the CT value of 18S (2-Dct).

### Measurement of intracellular Ca^2+^

Myocytes were loaded with 3 µM Fura-2/AM in physiological saline solution for 1 h at 25 °C. Then, the cells were allowed to settle into a perfusion chamber mounted on a Nikon inverted microscope (Diaphot 200; Minato-ku, Tokyo, Japan). The cells that adhered to the glass were continuously perfused at a rate of 2.5 ml/min with physiological saline solution (37 °C, equilibrated with 95% O_2_ and 5% CO_2_, pH = 7.4) containing 2 mM Ca^2+^. Cells were excited by alternating pulses of 340- and 380-nm-wavelength light, and the light emitted at 510 nm was measured using a PTI microphotometer (Photon Technology International, Princeton, NJ, USA). The cells in the field were removed before beginning the experiments. The background fluorescence was automatically subtracted by removing cells from the field. The fluorescence acquisition rate was 0.5 s. The intracellular Ca^2+^ concentration was calculated according to the Grynkiewicz et al. [21] formula. The Kd of Fura-2 was assumed to be 386 nM [22]. The mean 340/380 fluorescence ratios corresponding to Rmax and Rmin were determined by exposing the cells to 10 mM Ca^2+^ in the presence of 10 µM ionomycin and to Ca^2+^-free phosphate saline solution with 1.11 mM EGTA, respectively. Rmax was 11.7, and Rmin was 0.5. The fluorescence ratio resulting from 380 nm light excitation in Ca^2+^-free solution and Ca^2+^-saturated cells (ß) was 7.5.

### Reduced glutathione (GSH) measurement

Isolated myocytes were rapidly homogenized, and 100 mg of pelleted cells were mixed with 300 μl of 5% saline solution. The samples were centrifuged at 12,000×*g* at 4 °C for 20 min. The supernatant was collected, and quantitative measurement of GSH in the supernatant was performed with the GSH assay kit (ab235670, Abcam, USA). This kit utilizes a specific enzymatic cycling method in the presence of GSH and a fluorophore. The reduction of the fluorophore produces a stable fluorescent product, the fluorescence of which is directly proportional to the amount of GSH in the sample and can be tracked kinetically (Ex/Em = 535/587 nm).

### Automated morphometry analysis in lung tissues

The left lung lobe was fixed by manually perfusing it with 10% neutral buffered formaldehyde solution. The lung fragments were cut and embedded in paraffin, and 4 µm-thick lung sections were stained with Masson trichrome stain. The areas containing lamina propria and smooth muscle (µm^2^) were determined through the use of automated morphometry (Qwin, Leica Microsystems Imaging Solutions, Cambridge, UK). All measurements were conducted in six bronchi and six bronchioles chosen at random from each animal. The data were adjusted according to the length of the corresponding basement membrane, and their average value was considered the final result. Bronchi and bronchioles were identified by the presence or absence of cartilage in the airway wall, respectively.

### Drugs and reagents

Ovalbumin (chicken egg albumin grade II), Fura-2/AM, brefeldin-A, caffeine, cyclopiazonic acid, EGTA, ionomycin, papain, dispase II, cysteine, histamine dihydrochloride, and all salts and stains used for microscopy were purchased from Sigma Chemical Co. (St. Louis, MO, USA). Aluminium hydroxide was purchased from J.T. Baker (Phillipsburg, NJ, USA). Pentobarbital sodium was acquired from Pfizer (Toluca, Mexico). Fura-2/AM and CPA were dissolved in dimethyl sulfoxide (final concentration 0.025%). In the control experiments, dimethyl sulfoxide had no effect.

### Statistical analysis

The Δ baseline Bi represented the difference between the reinforcement value and the last baseline Bi value obtained before challenge. Airway responsiveness to histamine was evaluated according to the provocative dose 200% (PD_200_), i.e., the interpolated histamine dose that caused a three-fold increase in comparison to the basal Bi. Changes in histamine responsiveness induced by saline or antigenic challenges were evaluated by means of a paired Student’s t-test and by dividing the PD_200_ observed after challenge by the PD_200_ value observed before challenge (PD_200_ ratio). For multiple group comparisons, repeated measures ANOVA followed by Dunnett’s tests was used. Associations between TGF-β1, GSH or SERCA-2b and changes in lung function were assessed through Spearman correlation coefficient analysis. Statistical significance was indicated by a two-tailed *P* < 0.05. The data in the text and figures are expressed as the mean ± SEM.

## Results

### Antigen-induced lung functional changes

**Fig. 2:**
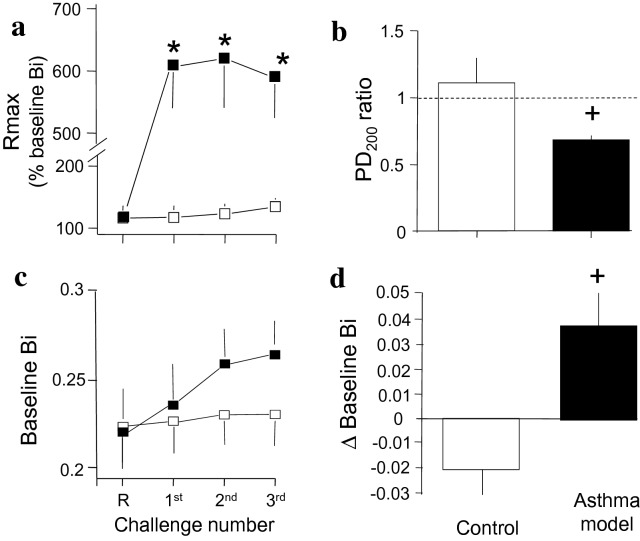
Antigen-induced changes in lung function in guinea pigs. **a** Average maximum (Rmax) bronchoobstructive index (Bi) induced by indirect (ovalbumin antigen) provocation. **b** The PD_200_ ratio resulting from direct (histamine) provocation was determined according to the PD_200_ value observed after antigen challenge divided by the PD_200_ value determined before challenge. **c** The average intrinsic baseline Bi obtained before indirect and direct provocation. **d** Difference in the baseline Bi (Δ baseline Bi) obtained after reinforcement (R) and the last challenge with saline (white bar, control) or antigen (black bar, asthma model). Values correspond to challenge with saline (white square) and antigen (black squares) in guinea pigs. *P < 0.05 compared with the control according to repeated measures ANOVA followed by Dunnett’s test. ^+^*P* < 0.05 compared with the control (unpaired Student’s t-test). The dotted line in B shows the border between hypo- and hyperresponsiveness

After sensitization and reinforcement with antigen, ovalbumin challenge induced transient airway obstruction. The average maximal obstructive response (Rmax) observed during the first through the third antigen challenge was higher in the guinea pig asthma model than in the control group (*P* < 0.01; *n* = 6 and 9 for the control and asthma model groups, respectively; Fig. 2a). Airway responsiveness was evaluated by comparing histamine responses before and after the last challenge. The PD_200_ ratio in the control group was close to 1, meaning that the PD_200_ value after saline challenge was similar to the initial challenge PD_200_ value. In the asthma model, the PD_200_ after antigen challenge was lower than the PD_200_ obtained before challenge, and the PD_200_ ratio was significantly lower than that obtained in the control group (*P* < 0.05; *n* = 6 and 9 for the control and asthma model groups, respectively; Fig. 2b). The changes in the airway baseline Bi (intrinsic tone) determined before direct or indirect airway provocation are shown in Fig. 2c. During the challenges, the Δ baseline Bi in the asthma model was increased in comparison that in the controls (*P* < 0.05; *n* = 6 and 9 for the control and asthma model groups, respectively; Fig. 2d). The PD_200_ ratio showed an inverse association with airway obstruction (*r* = − 0.5, *P* = 0.04), meaning that an increased AHR resulted in an increased Rmax. No correlation was observed between the extent of antigen-induced obstruction and the PD_200_ ratio according to Δ baseline Bi (*r* = 0.14, *P* = 0.3, and *r* = 0.18, *P* = 0.25, respectively).

### Relationship of GSH levels in myocytes with functional changes

**Fig. 3:**
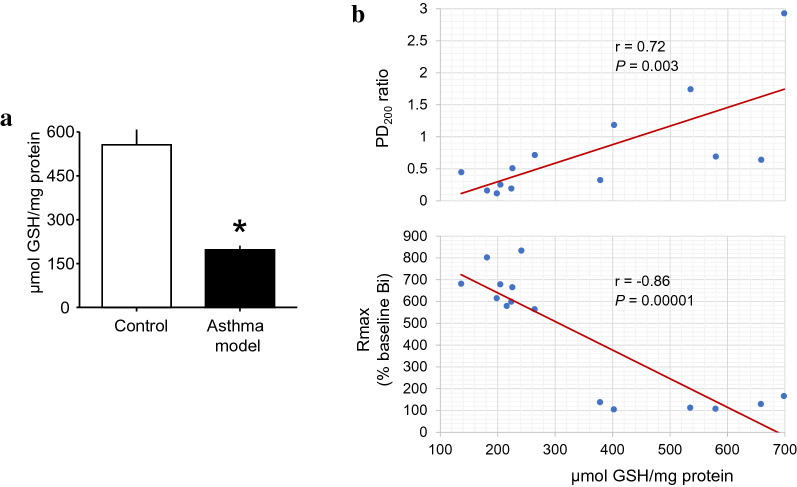
GSH changes in isolated airway smooth muscle cells from a guinea pig asthma model. **a** Levels of GSH in isolated myocytes. Scatter graphs showing that GSH levels were correlated (**b**) directly with changes in the PD_200_ ratio (upper panel) and inversely with Rmax values (lower panel). *P = 0.0064 compared with the control (unpaired Student’s t-test). *r* = Spearman correlation coefficient; *P* = paired Student’s t-test

The levels of GSH were decreased in asthma model guinea pigs in comparison with those in controls (*P* = 0.0064, *n* = 6; Fig. 3a). The GSH levels showed a direct correlation with the PD_200_ ratio (*P* = 0.003, *n* = 13) and an inverse correlation with Rmax (*P* = 0.00001, *n* = 15; Fig. 3b), implying that the greater the GSH level was, the lower the levels of antigen-induced AHR and airway obstruction were. The GSH levels were not correlated with Δ baseline Bi (*r* = 0.04, *P* = 0.48, *n* = 12).

### Relationship of IL-13 and TGF-β1 expression in myocytes with airway functional and structural changes

**Fig. 4:**
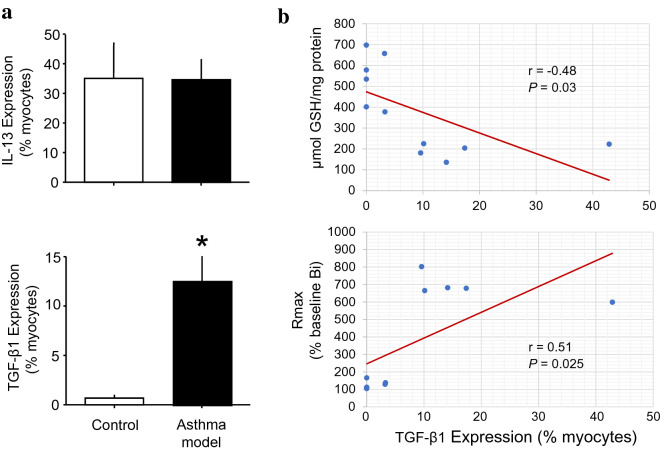
Number of isolated airway myocytes that expressed IL-13 and TGF-β1 measured by flow cytometry. **a** Bars represent the mean + SEM. **b** Scatter graphs showing that the number of isolated airway myocytes that expressed TGF-β1 was correlated inversely with GSH levels in myocytes (upper panel) and directly with Rmax (lower panel). *P < 0.05 compared with the control (unpaired Student’s t-test). *r* = Spearman correlation coefficient; *P* = paired Student’s t-test

In isolated tracheal myocytes, the number of myocytes that expressed IL-13 did not change between groups (Fig. 4a, upper panel). The number of cells that expressed TGF-β1 was increased in the asthma model compared with that in the control group (*P* < 0.05; *n* = 6 each group; Fig. 4a, lower panel). GSH levels were inversely correlated with the expression of TGF-β1 in myocytes (*P* = 0.03, *n* = 12; Fig. 4b, upper panel). No correlation was observed between the number of cells that expressed TGF-β1 and the PD_200_ ratio or Δ baseline Bi (TGF-β1 vs PD_200_ ratio and Δ baseline Bi: *r* = − 0.42, *P* = 0.07, and *r* = 0.13, P = 0.32, respectively), but a positive correlation was observed between the number of cells that expressed TGF-β1 and Rmax (*P* < 0.025, *n* = 12; Fig. 4b, lower panel). The number of cells that expressed IL-13 was not correlated with the PD_200_ ratio, Δ baseline Bi or Rmax.

**Fig. 5:**
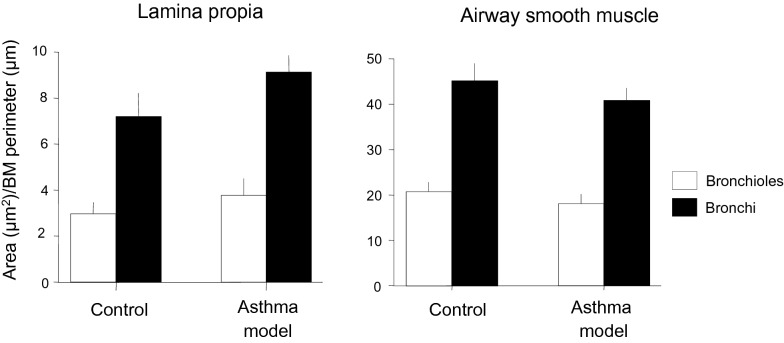
Airway wall areas. Data were adjusted according to the length of the corresponding basement membrane (BM). Bars represent the mean ± SEM

The increase in the number of myocytes that expressed TGF-β1 did not affect the mass of the airway wall, indicating that the increase in TGF-β1 was not able to produce fibrosis or changes in the airway smooth muscle mass (*n* = 6 each, bronchi and bronchioles; Fig. 5). No correlation was observed between the mass of the airway wall and TGF-β1 levels or changes in lung function according to Δ baseline Bi, the PD_200_ ratio or Rmax.

### Relationship of SERCA2b expression in myocytes with functional changes

**Fig. 6:**
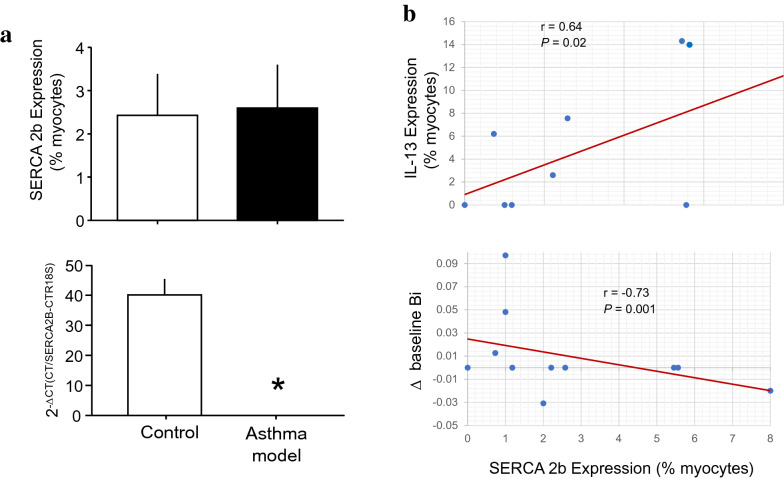
SERCA2b expression in asthma model guinea pigs. **a** Number of isolated airway myocytes that expressed SERCA2b determined by flow cytometry (upper panel). *SERCA2B* gene expression measured by RT-PCR (lower panel). Scatter graphs showing that the number of myocytes that expressed SERCA2b was correlated (**b**, upper panel) directly with the number of myocytes that expressed IL-13 and (**b**, lower panel) inversely with the changes (Δ) in the baseline bronchoobstructive index (Bi). *P = 0.00001 compared with the control (unpaired Student’s t-test). *r* = Spearman correlation coefficient; *P* = paired Student’s t-test

The number of ASM cells that expressed SERCA2b did not change between groups; nevertheless, the RT-PCR results showed a decrease in *SERCA2B* gene expression in tracheal smooth muscle in the asthma model group in comparison with that in controls (*P* = 0.00001, *n* = 6 each group; Fig. 6a). The number of myocytes that expressed SERCA2b showed a direct correlation with the number of myocytes that expressed IL-13 (*P* = 0.02, *n* = 9; Fig. 6b, upper panel). An inverse relationship was observed between Δ baseline Bi and the number of myocytes that expressed SERCA2b (*P* = 0.001, *n* = 11; Fig. 6b, lower panel). No significant correlation was observed between the number of myocytes that expressed SERCA2b and the PD_200_ ratio, Rmax, or TGF-β1 and GSH levels.

**Fig. 7:**
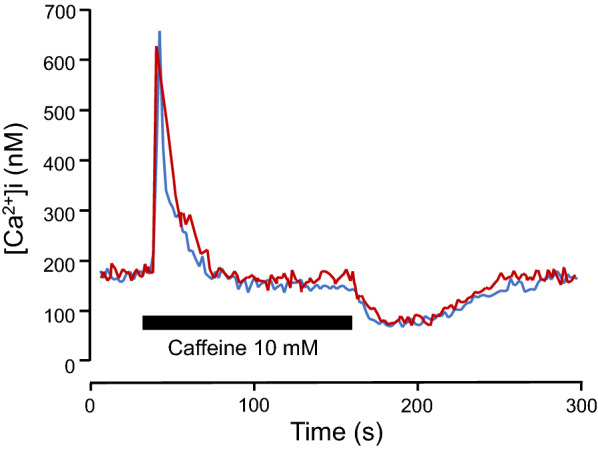
Representative recording showing the changes in intracellular Ca^2+^ levels ([Ca^2+^]_i_) induced by caffeine (10 mM) in myocytes isolated from control (red) and asthma model guinea pigs (blue). The withdrawal of caffeine induced a Ca^2+^ undershoot

To evaluate the rate of reuptake of Ca^2+^ by SERCA, the Ca^2+^ in the sarcoplasmic reticulum in myocytes was depleted by incubating the cells with 10 mM caffeine for 2 min. The refilling of the sarcoplasmic reticulum was allowed to produce a Ca^2+^ undershoot after caffeine withdrawal (Fig. 7). In the control and asthma model myocytes, the basal Ca^2+^ levels were comparable (137 ± 7.6 nM, *n* = 6; 139 ± 6.8 nM, *n* = 5). The rate of Ca^2+^ decrease during Ca^2+^ undershoot in myocytes from control and asthma model guinea pigs was similar (4.6 ± 0.6 nM/s, *n* = 6 and 4.3 ± 0.5 nM/s, *n* = 5, respectively; Fig. 7), suggesting that the reuptake of Ca^2+^ by SERCA was not altered in the asthma model.

## Discussion

Asthma is a heterogeneous disorder characterized by pathophysiological alterations of airways that are responsible for the symptoms of the disease. In this study, we observed that the guinea pigs that showed a greater response to direct ASM stimulation with histamine and, consequently, a high AHR also presented greater airway obstruction in response to indirect challenge with antigen, suggesting that the behaviour of ASM in the asthma model reflected a hypercontractile phenotype. It was previously shown that an increase in ASM contractile capacity induced an increase in airway tone and enhanced airway responsiveness [4]. Our results showed that the change in the baseline intrinsic tone did not show any association with the extent of airway obstruction induced by either direct or indirect provocation. Indeed, our results suggest that in the guinea pig asthma model, the mechanism involved in the change in the baseline intrinsic tone was different from that that induced hypercontractility. Therefore, the hypercontractile phenotype of ASM was associated with decreased levels of GSH, highlighting the importance of redox balance to ASM cell behaviour.

Glutathione is considered the principal cellular redox buffer molecule. In a healthy cell, 98% of glutathione is found in its reduced form, GSH. Lung GSH depletion has been observed in an allergic asthma model in guinea pigs [14]. This depletion could play an important role in anaphylaxis. In mast cells, GSH is capable of abating histamine release [23], while in isolated tracheal rings, GSH reduced the contraction induced by histamine compared with that induced by carbachol or 5-hydroxytryptamine [24]. It is known that the response to antigen provocation in guinea pigs is mainly induced by the release of histamine [25]. Therefore, the increase in the direct or indirect histamine response observed in our study can be explained by the reduction of GSH in ASM cells. In addition, the reduction of GSH could be produced by TGF-β1 because it is known that TGF-β1 has effects on redox balance by depleting glutathione levels [26].

TGF-β1 is a pleiotropic cytokine with immunosuppressive, pro- and anti-inflammatory and fibrogenic properties [27]. Due to the role of this cytokine in fibroblast recruitment and proliferation, extracellular matrix synthesis and inhibition of matrix metalloproteinases, TGF-β1 is considered to play a key role in airway remodelling and consequent airway narrowing [28]. In our study, although the percentage of ASM cells that expressed TGF-β1 was increased in asthma model guinea pigs, we did not observe evidence of structural remodelling, such as changes in ASM mass or the development of subepithelial fibrosis. In addition, TGF-β1 can contribute to bronchoconstriction by increasing the expression of contractile proteins [29] and enhancing force generation, agonist-induced contraction and excitation–contraction coupling by ASM [30]. This can explain the relationship between antigen-induced airway obstruction and TGF-β1 levels observed in our study; paradoxically, airway responsiveness and TGF-β1 levels were not associated, suggesting that there are some mechanisms involved in indirect challenge with antigen that are affected by TGF-β1 that are not involved in the direct stimulation of ASM by histamine. In agreement with this observation, it has been shown that the role of TGF-β1 in airway responsiveness is controversial [30, 31]. In addition, TGF-β1 is capable of directing immune responses against antigens, inducing allergic responses [32]. Thus, the main physiopathological role of TGF-β1 in ASM cells seems to be the intensification of allergic responses to antigens.

An intracellular Ca^2+^ increase is necessary for ASM contraction. A key regulator of intracellular Ca^2+^ homeostasis in ASM is SERCA2b. The role of SERCA in ASM is dual: it causes muscle relaxation by lowering cytosolic Ca^2+^ levels and re-establishes the sarcoplasmic reticulum Ca^2+^ supply, which is necessary for muscle contraction; therefore, alterations in SERCA have been implicated in many types of pathological processes. It has been reported that the SERCA2b protein is specifically expressed in both native and cultured ASMs obtained from endobronchial biopsies of patients with asthma. SERCA activity can be influenced by various molecules, such as GSH. A steady supply of GSH results in *S*-glutathionylation of SERCA, increasing its activity [33]. In the heart, the increase in the expression of SERCA1a, the main SERCA isotype in cardiomyocytes, enhances myocyte contractility in response to caffeine in a gene-dose-dependent manner and alters myocyte shortening due to increased SERCA activity and Ca^2+^ buffering [34]. In contrast, decreasing SERCA levels in vascular smooth muscle will produce intracellular Ca^2+^ oscillations [35]. Human ASM cells from asthma patients did not show changes in baseline intracellular Ca^2+^ levels or the expression of SERCA; nevertheless, asthma patients with ASM cells with an increased rate of intracellular Ca^2+^ clearance were shown to have decreased lung function [36]. Another study showed that SERCA expression is diminished in ASM from asthma patients in comparison with that in ASM from healthy subjects and that the extent of this effect was correlated with the severity of disease [16]. In line with this finding, we observed in our study that the levels of the SERCA2B gene were reduced in ASM from the asthma group; however, SERCA2b protein expression did not show changes between groups. Furthermore, we found that the intrinsic baseline tone had an inverse association with the percentage of myocytes that expressed SERCA2b, suggesting that the increase in tone could be produced by reduced buffering of Ca^2+^; nevertheless, the sarcoplasmic reticulum Ca^2+^ refilling rate was similar in myocytes from controls and asthma model guinea pigs, suggesting that the function of SERCA2b was not altered in asthma model guinea pigs and that subtle changes in SERCA2b expression are enough to induce changes in the intrinsic baseline tone.

IL-13 is a cytokine that has been related to the expression of SERCA and Ca^2+^ signalling. In murine ASM cells, IL-13 can induce Ca^2+^ signalling and contraction [37], while in human ASM cells, IL-13 can enhance Ca^2+^ responses after stimulation with histamine [38] and boost the contractility of airways via its autocrine effects on ASM [7]. In 2009, Sathish et al. [39] found that incubation of human ASM cells with IL-13 reduced the expression of SERCA2b, but in our study, we did not observe changes in the percentage of ASM cells that expressed IL-13 in the asthma model or the relationship of IL-13 expression with pathophysiological changes, although an association between IL-13 and SERCA2b in ASM cells was observed.

## Conclusion

Taken together, the experimental results described in this study suggest that ASM cells are phenotypically heterogeneous and that some phenotypes are associated with different effects during airway obstruction. Because of this, the asthma model revealed a hypercontractile ASM phenotype characterized by low GSH levels. This hypercontractile phenotype is important in the development of extrinsic obstruction that produces either direct or indirect ASM provocation. Interestingly, an ASM cell phenotype that produces high levels of TGF-β1 is predominantly characterised by extrinsic indirect obstruction induced by allergens. Finally, the intrinsic airway baseline tone is influenced by SERCA2b expression in ASM cells but not airway wall enlargement. The study of ASM cell phenotypes might be valuable for understanding the heterogeneity, variability and complexity of asthma.

## Data Availability

The data that support the findings of this study are available on request from the corresponding author upon reasonable request.

## Abbreviations

AHR: Airway hyperresponsiveness
ASM: Airway smooth muscle
Bi: Bronchoobstructive index
Δ baselineBi: Bi change compared to the airway baseline Bi
Ca^2^^+^: Calcium
ELISA: Enzyme-linked immunosorbent assay
Fura-2/AM: Fura-2 acetoxymethyl ester
GSH: Reduced glutathione
GSSG: Glutathione disulphide
IL-13: Interleukin-13
PD_200_: The interpolated histamine dose that caused a three-fold increase of the basal Bi
PD_200_ratio: PD_200_ observed after challenge by the PD_200_ value observed before challenge
PEP: Peak expiratory pressure
PIP: Peak inspiratory pressure
Rmax: Maximal obstructive response
Rt: Relaxation time
RT-PCR: Real-time polymerase chain reaction
SERCA: Sarco-endoplasmic Ca^2^^+^ ATPase
Te: Expiratory time
TGF-β1: Transforming growth factor-β1
